# Balance Training-Related Changes in Intracortical Inhibition and Symptom Severity in a Patient with Chronic Neuropathic Pain: A Single-Case Study

**DOI:** 10.3390/brainsci16020203

**Published:** 2026-02-09

**Authors:** Wolfgang Taube, Naima Mory, Franziska Peier, Michael Mouthon, Joelle N. Chabwine, Benedikt Lauber

**Affiliations:** 1 Department of Neuroscience and Movement Science, University of Fribourg, 1700 Fribourg, Switzerland; franziska.peier@unifr.ch (F.P.); benedikt.lauber@unifr.ch (B.L.); 2 Laboratory for Neurorehabilitation Science, Medicine Section, University of Fribourg, 1700 Fribourg, Switzerland; naimamory@gmail.com (N.M.); michael.mouthon@unifr.ch (M.M.); joelle.chabwine@unifr.ch (J.N.C.); 3 Research Division, Department of Internal Medicine and Specialties, Fribourg Hospital, 1752 Villars-sur-Glâne, Switzerland; 4 Division of Neurorehabilitation, Fribourg Hospital, 3280 Meyriez-Murten, Switzerland

**Keywords:** chronic neuropathic pain, GABA, intracortical inhibition, transcranial magnetic stimulation, balance training, sleep

## Abstract

**Highlights:**

**What are the main findings?**

- Several weeks of balance training reduced chronic neuropathic pain and improved sleep and well-being.
- GABA-mediated intracortical inhibition, measured by transcranial magnetic stimulation, was enhanced.

**What are the implications of the main findings?**

- Targeted physical activity can be used to improve pain perception.
- The most likely mechanism is upregulation of GABAergic inhibitory circuits in cortical regions.

**Abstract:**

**Background/Objectives**: It is widely recognized that malfunctions in the GABAergic system can be one of the underlying mechanisms in chronic pain. However, the use of GABAergic drugs to improve pain perception has strong and unwanted side effects, particularly in terms of sedation. Therefore, the present exploratory single-case study tested an alternative treatment using balance training to upregulate the GABAergic system in a 62-year-old patient with widespread chronic pain. Previously, balance training was shown to increase short-interval intracortical inhibition (SICI), a neurophysiological marker commonly associated with GABA-mediated intracortical inhibition, as assessed using paired-pulse transcranial magnetic stimulation (TMS), in healthy young and older adults. Therefore, we hypothesized that the balance-training-induced increase in GABA_A_-related intracortical inhibition would alleviate pain and increase quality of life. **Methods**: After two baseline measures, the patient participated in two balance training periods of 4 weeks each, followed by two detraining phases of 2 months each. At baseline and after each intervention and each detraining, intracortical inhibition (i.e., SICI) as well as pain and ‘well-being’ (questionnaires) was assessed. **Results**: Our results demonstrated enhanced and better modulated intracortical inhibition after 4 weeks of balance training, which was in line with analgesia and improved sleep and mood scores. However, after the first detraining, all parameters went back to baseline. In a subsequent second period of 4 weeks of balance training, intracortical inhibition was again increased, even above the values of the first training period. Pain, sleep, and mood scores were also further improved. After the second detraining period, all values dropped back close to their baseline values. **Conclusions**: The findings support the assumption that the GABAergic system is highly relevant in the processing and perception of pain. More importantly, our results suggest the possibility that balance training may be an effective way not only to upregulate intracortical inhibition but also to alleviate pain and improve well-being in patients with unspecific chronic pain.

## 1. Introduction

There is growing evidence that chronic pain (CP) is associated with hyperexcitability in nociceptive pathways and is mediated at least partly by decreased inhibitory (GABAergic) input [1,2,3,4]. In line with this, reduced cortical GABA, the main inhibitory neurotransmitter in the brain, has been observed in CP patients by means of magnetic resonance spectroscopy [5]. When using paired-pulse transcranial magnetic stimulation (TMS) to assess short-interval intracortical inhibition (SICI), a marker of GABA_A_ activity in the motor cortex [6,7], SICI was reported to be reduced in patients with neuropathic pain compared to healthy controls without pain [8,9]. Similarly, intracortical inhibition was lower in patients with fibromyalgia in comparison to healthy subjects [10]. Interestingly, subthreshold repetitive TMS (rTMS) over the primary motor cortex (M1) was shown to induce analgesic effects in patients with chronic widespread pain in the context of fibromyalgia [11]. At the same time, subthreshold rTMS is known to upregulate SICI [12]. In line with this, Lefaucheur et al. demonstrated that the reduced intracortical inhibition in chronic neuropathic pain patients could be upregulated by a subthreshold rTMS treatment over M1 [13]. At the same time, enhanced intracortical inhibition was associated with reduction in pain perception. Consequently, these authors proposed that the rTMS-induced analgesic effects may arise, at least partly, from the restoration of defective intracortical inhibitory processes [13].

Apart from rTMS, pharmacological drugs can be used to influence the GABAergic system and reduce pain. However, GABAergic drugs have strong, negative side effects such as sedation and, therefore, are of limited utility [14]. Another much less investigated way to manipulate the inhibitory system is through physical exercise. Short-term learning/training studies indicate a general downregulation of the motor cortical inhibitory system irrespective of the type of physical activity/learning [15]. This may be expected, as the initial phase of motor learning is characterized by plastic neural changes that rely on the downregulation of inhibitory processes. For instance, animal experiments highlighted the importance of the transient suppression of local GABA_A_ inhibition to induce LTP-like processes [16], as the decrease in GABA promotes AMPA receptor-mediated plasticity [17]. Furthermore, in the early stages of plasticity, the decrease in GABAergic inhibition may promote ‘unmasking’ of existing horizontal connections, a mechanism that is thought to reinforce the rapid remodelling of motor representations [18,19]. Taking all this into account, it is not surprising that short-term studies investigating intracortical inhibitory control suggest a general decrease in SICI as a consequence of motor learning. However, longer-term studies demonstrate a more differentiated picture. Strength training over several weeks induces a decrease in inhibition [20,21,22], which is thought to increase neural drive and thus force and/or explosive strength. In contrast, training regimes incorporating coordinatively challenging balance exercises were shown to upregulate intracortical inhibition in healthy young [23,24,25] as well as elderly subjects [26]. Furthermore, in elderly people, it was demonstrated using magnetic resonance spectroscopy (MRS) that the amount of GABA in the sensorimotor cortex could be enhanced after 12 weeks of balance training [27]. In a recent review paper, it was proposed that training-induced changes in intracortical inhibition indicate an improved ‘ability to modulate GABAergic inhibition’, whereas the increase in GABA measured by MRS indicates an increased ‘capacity for inhibition’ [28]. Importantly, these studies demonstrate that it is possible to upregulate intracortical inhibition in the long term, even in populations with low levels of GABA-mediated intracortical inhibition such as elderly people.

The present exploratory single-case study therefore aimed to investigate the effects of balance training (each training block lasted for 4 weeks) as well as the effects of subsequent periods of detraining on intracortical inhibition in a 62-year-old patient with widespread CP. Intracortical inhibition was measured with a paired-pulse TMS paradigm that assesses GABA_A_-related inhibition. It was hypothesized that balance training would increase intracortical inhibition, enhance the modulation of intracortical inhibition measured in different postural conditions, and improve pain, sleep, and depression scores (i.e., general well-being).

## 2. Materials and Methods

*Study participant*: A 62-year-old male patient who has had widespread chronic pain since 2019 participated in this study. At the start of the study, the patient suffered from widespread and burning pain with predominance at the upper and lower extremities. Pain occurred spontaneously but was exacerbated at night just before sleeping by cold and friction, especially with materials such as aluminum, animal fur, hairs, and all sorts of textiles, which also evoked strange and unpleasant sensations. Associated symptoms were generalized electric shock sensations, pins and needles paresthesia, and skin colour changes (whitish or blueish) in the extremities.

The clinical workup revealed neuropathic features (10/39 on the Small Fiber Neuropathy and Symptom Inventory Questionnaire [29] score), namely a superimposed sensory deficit configuration recalling polyneuropathy (stocking and glove hypo/anesthesia, abolished tendon reflexes). The diagnostic criteria for fibromyalgia [30] were not fulfilled despite the widespread pattern of pain.

*Balance performance*: Changes in postural control were assessed on a moveable platform (Posturomed^TM^, Muenchweiler, Germany), allowing for translational movements in the transversal plane (for technical details, see [31]). During three 15 s trials, anterior–posterior and medio-lateral sway paths were recorded by joystick potentiometers connected to the moveable platform. The cumulative sway path of the three trials was recorded for a bipedal stance, which comprised a single-leg stance on the right and left legs with arms akimbo. Trials in which the participant had to hold on to a handrail or had to step off the device were discarded and repeated.

*EMG*: EMG recordings were obtained from the tibialis anterior muscle (TA) and m. soleus of the right leg. After skin preparation (shaving, disinfecting), bipolar surface electrodes (Blue sensor P, Ambu, Bad Nauheim, Germany) were attached to the skin longitudinally above the muscle belly (2 cm inter-electrode distance) according to SENIAM guidelines. The reference electrode was placed on the tibial plateau. EMG signals were amplified (×1000), bandpass-filtered (10–1000 Hz), and sampled at 4 kHz.

*Transcranial magnetic stimulation (TMS)*: Transcranial magnetic stimuli were applied over the left hemisphere motor cortex via a MagPro X100 with a MagOption stimulator. A 95 mm focal ‘butterfly-shaped’ coil (D-B80, both MagVenture A/S, Farum, Denmark) was used with the handle of the coil pointing backwards, and the stimulator was programmed to induce a posterior–anterior current flow in the motor cortex. The cable of the coil was fixed to a pulley system that was attached to the ceiling to minimize cable forces. The initial stimulation point was set 0.5 cm anterior to the vertex and over the midline. Subsequently, the final coil position was established by moving the coil anterior and left from the vertex, during which the size of the motor-evoked potential in the TA was permanently checked. The final position (hotspot for the TA) was marked on the scalp. The coil was held by an experienced operator, and the position was constantly monitored. We did not use a helmet like in many of our previous studies due to the relatively high pressure associated with this system. This would have caused too much pain for the patient.

*Resting and active motor thresholds*: The resting motor threshold (MT) was assessed during relaxed sitting. The MT was defined as the stimulation intensity to evoke MEPs larger than 100 μV in six out of ten consecutive trials in the TA. The active motor threshold (aMT) was assessed during different standing conditions in order to counteract changes in excitability and to ensure appropriate stimulation intensities to activate the inhibitory network in the same way as the MT (i.e., MEPs larger than 100 μV in six out of ten consecutive trials).

*Short-interval intracortical inhibition (SICI)*: Intracortical inhibition was tested using a paired-pulse TMS protocol where the suprathreshold single TMS pulse at 1.2 MT was preceded by the subthreshold (0.8 MT) TMS pulse by 2.5 ms [24]. While the first pulse serves as a conditioning pulse which is applied at intensities below the threshold to evoke an MEP (subthreshold TMS), the second pulse evokes a clearly visible MEP (suprathreshold MEP). The first pulse activates intracortical inhibitory interneurons, which then reduce the MEP amplitude of the second pulse, a phenomenon known as short-interval intracortical inhibition (SICI). The peak-to-peak amplitude of the conditioned MEP (paired-pulse TMS) is then compared to the unconditioned MEP (single pulse TMS). There is convincing evidence that SICI is a cortical phenomenon [7,32,33] and relies on GABA_A_ergic mechanisms [6].

In the present study, SICI was tested during (a) sitting, (b) upright stance, and (c) balancing on a foam cushion (AIREX). For each task, 20 single (unconditioned) and 20 paired pulse (conditioned) stimuli were recorded. According to previous research in healthy adults [24], it was expected that inhibition would be modulated across conditions with the highest levels of inhibition during sitting (as the muscles are inactive and should remain inactive) and the lowest inhibition during balancing on foam (requiring strong cortical involvement). Because of the changes in the level of muscle activation from rest to activity, the stimulation intensity was adjusted for each condition (see also ‘Resting and active motor thresholds’).

*Gabapentin*: It should be noted that the patient was treated with several analgesic treatments, including gabapentin. Due to pain exacerbation during the period between the baseline evaluation and balance training initiation (4 weeks from BASE to PRE1), the gabapentin dose was increased from 800 mg to 2000 mg per day.

*Assessment of pain and pain-induced interference*: Pain intensity was assessed using a visual analogue scale (VAS) at each assessment. The patient scored his level of pain on a scale ranging from 0 to 10, with 0 being ‘none’ and 10 being ‘most severe’. Pain interference was derived from the Brief Pain Inventory (BPI), rated on a scale from 0 to 10 indicating how pain interferes with (a) general activity, (b) mood, (c) walking, (d) normal work (inside and outside the home), (e) relations with other people, (f) sleep, and (g) enjoyment of life.

*Assessment of anxiety, depression, and insomnia*: Anxiety, depression, and insomnia were evaluated using standard questionnaires, namely the Hospital Anxiety and Depression Scale (HADanx, HADdep) and Insomnia Severity Index (ISI), at all measurement points (baseline, Pre1, Post1, Ret/Pre2, Post2, and Ret2).

*Balance training interventions*: The balance training was performed three times per week over a 4-week period. After a 15 min warm-up, the patient had to stand on different instable devices such as a two-dimensional swinging platform, wobbling boards, spinning tops, and soft mats. Each training exercise consisted of 3–6 sets with 30 s balancing on each of the devices. This resulted in 12 trials per leg at the beginning of training and was then increased to 24 trials in the last week of the training [24]. Furthermore, task difficulty was progressively increased (standing with eyes closed, catching a ball while balancing, etc.). There was a 30 s rest between trials and a 5 min break between sets. The patient stood barefoot on one leg on the devices. The training ended with a 15 min cooldown.

**Figure 1:**
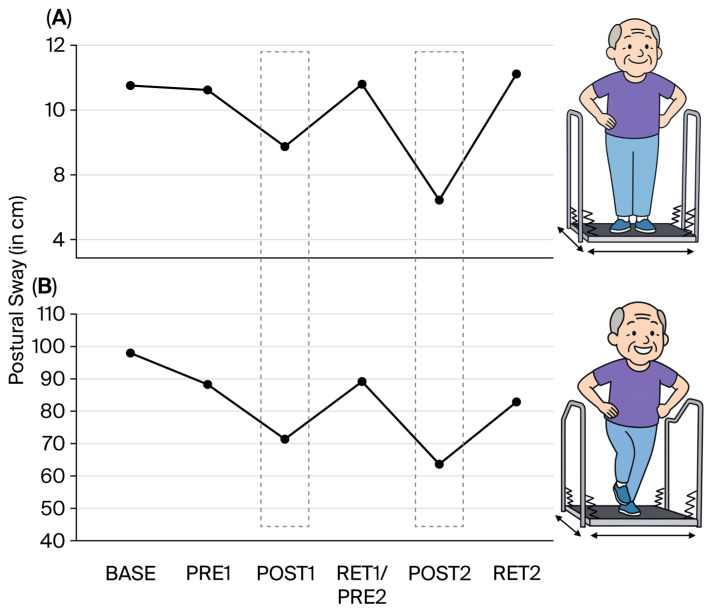
Changes in balance performance on the Posturomed. (**A**) indicates changes in postural sway measured in the bipedal stance on the free-swinging platform (i.e., Posturomed), whereas (**B**) displays the added sway path of the left and right single-leg stance on the Posturomed. It can be seen that after the balance learning interventions, the sway path was reduced (grey boxes). However, detraining always resulted in reduced performance, resulting in values close to baseline values for both (**A**) and (**B**).

*Data analysis*: Balance performance was analyzed by taking the best two trials out of the three recorded trials for each of the following conditions: (a) bipedal stance and (b) single-leg stance (sway paths of the right and left legs were added together). The cumulative sway paths were assessed at each measurement point (baseline, Pre1, Post1, Ret/Pre2, Post2, Ret2; see Figure 1).

SICI was analyzed for the three different conditions (sitting, standing, standing on unstable ground (AIREX)) by computing the peak-to-peak amplitudes of the motor-evoked potentials (MEPs) elicited by single (*n* = 20) and paired-pulse stimulation (*n* = 20). In the final analysis, the amplitudes of SICI were expressed as a percentage of inhibition using the following formula: 100 − (conditioned MEP/test MEP × 100).

## 3. Results

**Figure 2:**
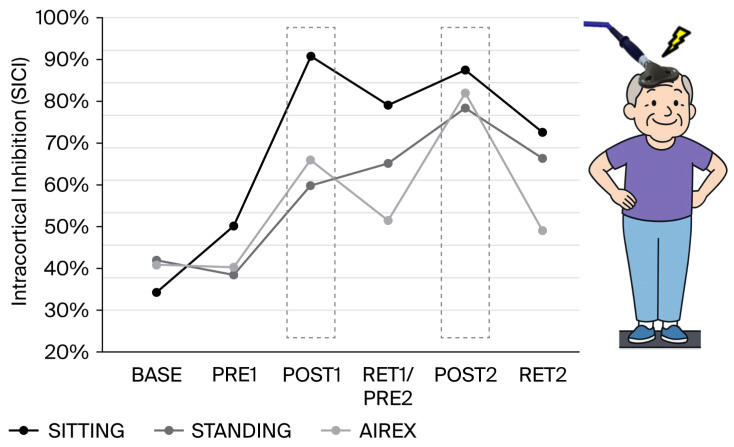
Changes in short-interval intracortical inhibition (SICI) in different conditions: (a) sitting, (b) standing, and (c) standing on unstable ground (Airex mat). It can be seen that inhibition was very low at baseline and was not modulated across conditions at this time. After balance training, inhibition was increased (grey dashed boxes). In Post1, modulation across conditions can be seen, with the highest inhibition during ‘sitting’ and lower inhibition during the more challenging ‘standing’ tasks. Detraining led to dis-inhibition, but values remained consistently higher than at baseline.

**Figure 3:**
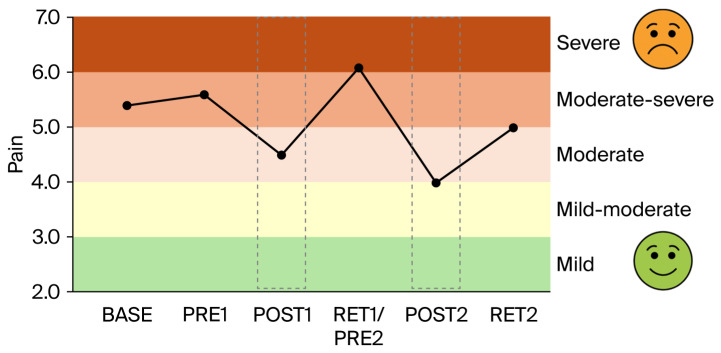
Score related to pain. Out of a maximum score of 10 (worst imaginable pain), the patient started with constant moderate-to-severe pain levels. Only after balance training interventions (first and second grey dashed boxes) were these scores reduced to moderate and mild–moderate, respectively.

**Figure 4:**
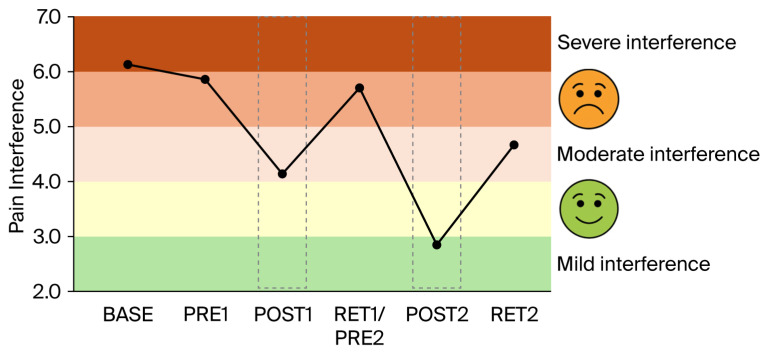
Score related to pain interference. Pain had a constant and severe effect on the patient’s general activities. Only after the second round of 4-week balance training (see POST2; second grey dashed box) did pain interference drop to 3 out of 10, indicating only mild interference in everyday life.

**Figure 5:**
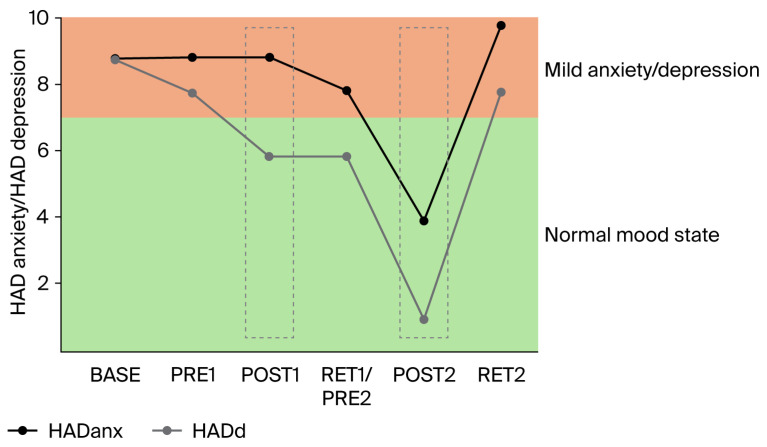
Scores related to anxiety and depression. Scores were determined using the HAD scales (maximum of 21 points). Both anxiety (HADanx, black dots) and depression (HADd, grey dots) levels changed most dramatically after the second 4-week balance training phase (see POST2; second grey dashed box).

**Figure 6:**
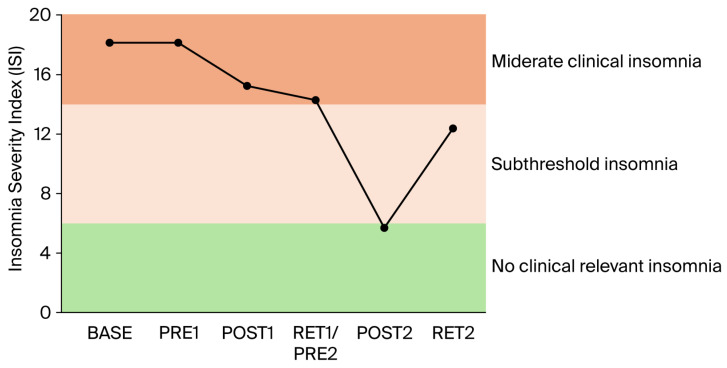
Sleep problems were determined using the Insomnia Severity Index (ISI). The patients started with 19 points out of 28, indicating moderate clinical insomnia (severe clinical insomnia is classified in the range from 22 to 28 points). After the first 4-week balance training, the score dropped to 16 but was still in the same range (i.e., moderate clinical insomnia). Only after the second 4-week balance training intervention (POST2) did the score reduce to 6 (i.e., no clinically relevant insomnia).

*Gabapentin*: The increase in gabapentin (from 800 to 2000 mg per day) between BASE and PRE1 (i.e., 4 weeks) had no or only minimal effects on balance performance (Figure 1A,B), intracortical inhibition (Figure 2), pain intensity (VAS; Figure 3), pain interference (Figure 4), anxiety and depression (Figure 5), and sleep (Figure 6).

*Postural control*: As expected, balance performance improved considerably after the first 4-week balance training intervention for both the bipedal (Figure 1A, post1) and single-leg stance (Figure 1B, post1). However, after 2 months of detraining, performance returned to baseline values (Ret1).

The second 4-week training intervention improved balance performance even more than the first intervention for both the bipedal and single-leg stance (Post2). For the bipedal stance, values again returned to baseline after 2 months of detraining (Ret2). For the more difficult single-leg stance condition (20 times more sway), a similar result was observed, but the values remained slightly better at Ret2 than at baseline.

Figure 1 does not display the number of trials that were disregarded due to using the handrail or stepping off the device. In the pre-training sessions, these disregarded trials occurred much more often (baseline: *n* = 13; pre1: *n* = 12; pre2: *n* = 13) than after balance training 1 (post1: *n* = 4) and balance training 2 (post2: *n* = 1).

*Short-interval intracortical inhibition*: The amount of SICI was not considerably changed by increasing the amount of gabapentin from 800 to 2000 mg per day (Figure 2). However, after the first 4-week balance training intervention, intracortical inhibition was increased in all three conditions. The increase was most pronounced for sitting and slightly weaker for standing upright and standing on the AIREX mat. After 2 months of detraining, SICI was slightly reduced for the sitting and AIREX conditions but remained high for standing. After the second 4-week balance training intervention, SICI was enhanced again for each condition. The second phase of detraining resulted in reductions in SICI, but the amount of inhibition remained nevertheless considerably higher than at baseline.

*Clinical scores related to pain, pain interference, mood, and sleep*: The clinical scores related to pain (Figure 3), pain interference (Figure 4), mood (anxiety and depression; Figure 5), and sleep (Figure 6) demonstrated very similar patterns. The first 4-week balance training intervention had a positive impact (POST1), but more dramatic changes were seen in all parameters after the second 4-week intervention (POST2). Unfortunately, most parameters demonstrated equally rapid changes in the other direction during de-training (RET1 and RET2).

## 4. Discussion

The most important finding of the present exploratory single-case study is the reduction in pain and the increase in well-being (sleep, mood) after balance training, which was accompanied by increases in GABA-mediated intracortical inhibition.

The present study confirms previous observations that were reported after performing balance exercises over a longer period of time and provides new perspectives on how balance training may influence pain perception. Over ten years ago, it was already demonstrated that balance training can reduce pain [34]. In this early study, a cohort of patients with chronic neck pain trained with balance exercises for 5 weeks and demonstrated reduced pain perception and improved joint repositioning accuracy. Furthermore, the reduction in pain was correlated with the improved sensorimotor function of the cervical spine. Therefore, the authors speculated that balance training affected supraspinal (mainly cortical) structures that were responsible for both sensorimotor control and pain perception [34]. More recently, it was demonstrated that balance training can upregulate intracortical inhibition in healthy young adults [23]. This finding was confirmed in later studies in young [24,25] and older adults [26]. Furthermore, it was demonstrated that not only intracortical inhibition but also the amount of GABA in the sensorimotor cortex could be increased in elderly adults after several weeks of balance training [27]. The findings in the latter study, especially in terms of the elderly, are important, as they demonstrate that populations with low levels of GABA and reduced intracortical inhibition can actually upregulate these GABAergic inhibitory processes after balance training. This was also the case in the present single-case study, in which the patient started with a very low level of intracortical inhibition at baseline but showed upregulated inhibition after each period of four-week balance training (see Figure 2; grey dashed boxes). Furthermore, after balance training, modulating inhibition across conditions became apparent, with the highest level of inhibition during ‘sitting’ and lower levels of inhibition during the more challenging ‘standing’ tasks. This makes sense as during sitting, the activation of muscles is usually not necessary and, thus, their neural drive can be inhibited. With increased postural task difficulty, cortical inhibition was, however, reduced, which was in line with previous observations in healthy young and older adults [23]. It is important to note that such a task-dependent modulation of intracortical inhibition was not seen in the baseline measurements or in PRE1. Thus, increasing the dose of gabapentin from 800 g to 2000 g did not change the level and modulation of intracortical inhibition. It also did not affect the perception of pain or the sleep quality.

One of the most important findings of the present study is that the upregulation of intracortical inhibition was accompanied by reductions in pain perception (see Figure 3) and pain interference (see Figure 4). As we have highlighted in the introduction, GABAergic processes are very likely to play a role in how sensory inputs are processed and interpreted and how (much) pain is perceived. In different cohorts of CP patients, intracortical inhibition was shown to be reduced compared to healthy controls [8,9,10]. By contrast, the upregulation of SICI by means of subthreshold repetitive TMS (rTMS) [12] could reduce pain [11,13]. It was therefore assumed that rTMS may, at least partly, restore the defective intracortical inhibitory processes in chronic pain patients [13]. Balance training may have similar effects on the GABAergic system to rTMS but seems superior for several reasons. In rare cases, rTMS may trigger epileptic seizures [35,36]. The chance of such an adverse event is assumed to be higher if the patient—like most chronic pain patients—is undergoing treatment with drugs, which potentially lower the seizure threshold [35]. Another negative side effect is brain implant heating, such as aneurysm clips or stimulation electrodes. If the surrounding tissue is heated above 43 °C, this may result in irreversible brain tissue damage [37]. Aside from this, the strong magnetic currents may displace brain implants [35]. Further potential risk factors include hypomania induction, headache, local pain, and transient hearing changes (please see Table 1 in [35]). Although the chances of negative side effects are relatively small, rTMS treatment is unpleasant for the patient. Furthermore, TMS treatment is much more costly than balance training. Thus, balance training can offer an alternative with many advantages. Most importantly, balance training is not only effective in upregulating intracortical inhibition, but it also lowers the risk of falls [38,39]. This is especially important in patients with chronic pain, which is known to be associated with a higher risk of falling [40,41]. In addition, balance training has previously been shown to broadly improve the sensorimotor function of the lower extremities not only by ameliorating postural control but also by enhancing the rate of force development and jump performance, as well as reducing the risk of ankle sprains in healthy and previously injured athletes, and giving-way episodes in ACL-injured people (for review [42]). Furthermore, not only was the sensorimotor function of the lower extremities found to be positively influenced by balance training, but the sensorimotor function of the cervical spine was also improved and pain was alleviated in people with chronic neck pain after 5 weeks of balance training [34]. Thus, balance training offers many additional benefits in addition to potentially reducing chronic widespread pain. In this respect, balance training may also counteract other factors negatively influencing well-being in patients with chronic pain. For example, it is well known that sleep is greatly impaired in these patients [43]. Primary pharmacological treatments against sleep disturbances target the GABAergic system, enhancing GABA_A_ receptor activity to suppress wakefulness and promote sleep [44,45,46]. Thus, GABA is not only important to regulate the perception of pain but also plays an important role in sleep regulation. We recently showed that three months of balance training in older adults led to improved subjective sleep quality, which was associated with increased GABA levels in the sensorimotor cortex (assessed with magnetic resonance spectroscopy), while training-related upregulation of intracortical inhibition during sleep (assessed with paired-pulse TMS) correlated with better subjective sleep quality (Scherrer et al. submitted). The data of the present study confirm this finding. The patient started with sleep scores indicating ‘moderate clinical insomnia’, but sleep disturbances were reduced after each block of 4-week balance training, with the most dramatic change seen after the second block of intervention (see Figure 6). At the same time, depression and anxiety scores improved (see Figure 5).

On the downside, the present study also demonstrated that periods of detraining led to reoccurrence of pain and increases in depression, anxiety, and insomnia scores (see RET2 in Figure 3, Figure 4, Figure 5 and Figure 6). Thus, it appears that balance training must be performed either for longer periods of time or even continuously to counteract pain and pain-related impairments in well-being. It seems promising that the level of intracortical inhibition did not return to baseline after the second detraining phase. Thus, several periods of balance training may lead to a continuously upregulated inhibitory system. Longer-term studies with larger cohorts are needed to verify this assumption, as the principal limitation of the present study was that data were obtained from only a single patient. This was necessitated by COVID-19 restrictions in place at the time of assessment, which precluded cohort studies and multi-participant training involving individuals at risk. The potential placebo effect also warrants consideration, as the act of leaving home, receiving attention, and participating in a structured activity alone may lead to meaningful improvements in pain, mood, and overall well-being. However, this psycho-social component can hardly explain changes in balance performance and SICI.

## 5. Conclusions

The present single-case study demonstrated reduced pain perception and improved sleep and well-being after balance training, which was accompanied by increases in GABA-mediated intracortical inhibition. It is therefore proposed that balance training may be an effective tool in order to counteract GABAergic modulation in chronic neuropathic pain.

## Data Availability

The original contributions presented in this study are included in the article. Further inquiries can be directed to the corresponding author.

## Abbreviations

The following abbreviations are used in this manuscript:

GABA	Gamma-aminobutyric acid
TMS	Transcranial magnetic stimulation
rTMS	Repetitive transcranial magnetic stimulation
SICI	Short-interval intracortical inhibition
CP	Chronic pain
M1	Primary motor cortex
MRS	Magnetic resonance spectroscopy
TA	Tibialis anterior muscle
MT	Motor threshold
aMT	Active motor threshold
MEP	Motor evoked potential
VAS	Visual analogue scale
BPI	Brief Pain Inventory
HAD_anx/dep_	Hospital Anxiety and Depression Scale
ISI	Insomnia Severity Index
ACL	Anterior cruciate ligament
